# HTA Metro Map: a patient centred model for optimizing the decision making process

**DOI:** 10.3205/hta000132

**Published:** 2019-09-24

**Authors:** Marco Chiumente, Khalid M. Kamal, Hans-Peter Dauben, Rainer Riedel, Inaki Gutierrez-Ibarluzea

**Affiliations:** 1 Scientific Direction, Italian Society for Clinical Pharmacy and Therapeutics, Milan, Italy; 2 Division of Pharmaceutical, Administrative and Social Sciences, School of Pharmacy, Duquesne University, Pittsburgh, United States; 3 Institute of Health Economics & Medical Outcome Research, University of Applied Sciences RFH, Cologne, Germany; 4 Basque Foundation for Health innovation and Research (BIOEF), Barakaldo (Bizkaia), Basque Country, Spain

**Keywords:** health technology assessment, capacity building, decision making, patient-centred care, continuing medical education

## Abstract

Health Technology Assessment (HTA) is a systematic evaluation of a health technology, designed to appraise the direct or intended effects and indirect or unintended consequences of the technology with an overall goal of supporting informed decision making regarding the use of these health technologies in the healthcare system.

In this paper, we present fundamental HTA concepts and provide a conceptual framework that embraces the processes and outcomes required for integrated healthcare decision-making. The “HTA Metro Map” was designed to guide the user through the different areas on: where to use, what and whom to involve within the decision process. The map reflects the complexity and inter-connectedness of the different kind of healthcare services that need to work together to be able to efficiently deliver coordinated decisions at local, regional, national, and international levels. This tool may also serve as base for facilitating developments and improvements of the HTA structure worldwide.

The paper discusses the main features of the “HTA Metro Map” while reinforcing the key concepts underlying HTA‘s integrated approach. The first view of the map provides the several layers of complexity seen in HTA and the various lines within the map represent the main actors involved in the assessment processes. The map connections and crossings symbolize the interprofessional and interpersonal collaborations while the stations denote the knowledge, skills, experiences, and attitudes of each professionals as they interact within this framework. Every line represents a HTA stakeholder and the circular line in the centre represents the patient at the centre of the system. The zones, from social to community and hospital level, represent the need for integration from the perspective of health systems. The HTA Metro Map also has different dimensions depicted by the level of profoundness. Finally, the concepts of different healthcare stakeholder perspectives are introduced both in visual and temporal terms.

The “HTA Metro Map” is designed as a flexible model for easy adaptability and in accurately capturing the complexity inherent in any healthcare system. It is hoped that the map will assist different stakeholders to build network capacity, pool existing resources, and develop a more holistic vision that will result in a sustainable, efficient and collaborative decision-making process.

## Introduction

Health Technology Assessment (HTA) is an essential support tool in healthcare decision-making due to its strategic importance of bridging the discrepancies between limited healthcare resources, adoption of novel technologies and improving the appropriate use of implemented health technologies [1], [2]. There are several definitions of HTA, but the most common agreed definition is: “The systematic evaluation of the properties and effects of a health technology, addressing the direct and intended effects of this technology, as well as its indirect and unintended consequences, and aimed mainly at informing decision making regarding health technologies [3].” 

In most industrialized nations, the healthcare costs are increasing which has resulted in budget containment. The healthcare costs have been increasing due to a growth in technological innovations, increased cost of healthcare services, aging populations and changes in both patient expectations and in the use of healthcare services [4], [5].

Some authors have stated that simply eliminating the inefficiencies in the healthcare system may not result in healthcare savings [6]. Efforts are being made in implementing and sustaining evidence-based technologies that improve outcomes through robustly demonstrating theoretical value (randomized controlled trials, systematic reviews, meta-analysis), assessing the benefits of a technology in relation to its costs (health economic evaluations), evaluating the suitability to the context in which it will be implemented (social, ethical, legal, environmental and organizational analyses) and improving safety (regulatory approaches on national and international level). Bringing all these ideas together revealed that the simple scientific one-dimensional approach probably led to misunderstandings, which may not have been in the best interest of public health-related policies, as well as having the issue of not solving the intention of improving context related outcomes and cost containment within health care services. Given the challenges, in the 1970s, general technology assessment (TA) approaches led to a new transdisciplinary approach called Health Technology Assessment (HTA) [7]. 

Today the terminology HTA is often misused and reduced to either clinical evaluations or economic analysis without paying much attention to other factors that determine the efficient use and the real value of health technologies in different contexts. Very often there is a lack of engagement of different healthcare stakeholders as well as in relation to the expectation, the acceptance and use of a technology as the organizational daily practice issues (standards of care, professional competencies and context related clinical pathways) or the acceptance and affordability challenges among others [8].

Therefore, in order to capture the complexity of health technologies and related interventions and to address the methodological challenges that involve and include opinions from different stakeholders, HTA needs to be multidimensional and transdisciplinary.

Transdisciplinary describes the need to bring together different kind of scientific attitudes; questions and discussions are done in cooperative teams at the same time to gain the multidisciplinary issues of identifying questions and answering them not just from one disciplinary perspective. 

Transdisciplinary approaches, although strongly desired, could be challenging given the multifold and often contrasting needs of different stakeholders and a lack of framework that aligns their interests. A seemingly simple solution to enhancing the collaboration among healthcare professionals, aimed at providing an accurate and complete assessment of health technologies, is to work on building competencies that can be leveraged for providing a robust evaluation supporting the decision making process. Training and education in this area are still very heterogeneous, despite several international agencies having published teaching manuals and many universities offering master courses in HTA [9], [10].

To account for the complexity and completeness of healthcare information, people assessing a technology and preparing a decision should definitely consider and acknowledge the transdisciplinary, multi-stakeholder approach of HTA.

Based on HTA methods, the objectives of this proposal are:

- highlighting the multidimensionality/transdisciplinary nature of the approach for a better decision making process,
- integrating and viewing HTA core concepts within the decision making process,
- offering non HTA experts, as well as HTA experts, a simplified but correct access to find the right person, method and interaction within a specific question, regarding local requirements as well as healthcare profession.

## “HTA Metro Map”: fundamental concepts

### The meaning of the map 

Many models could help to show different concepts of HTA, but no single model had been able to combine all the requirements in a way that could still be handled and depicted in a sole frame.

**Figure 1:**
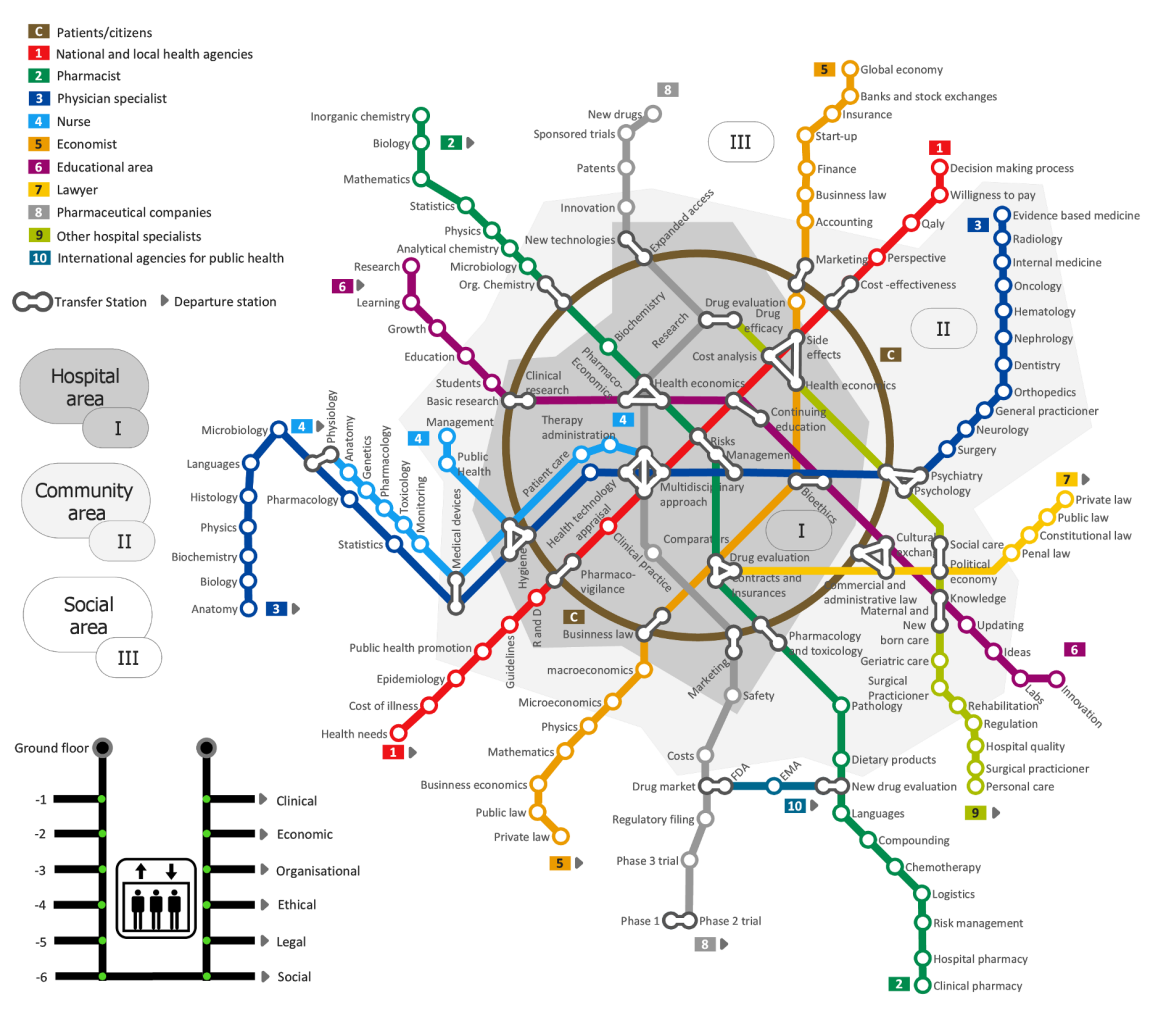
The “HTA Metro Map” provides a conceptual framework of immediate impact for capacity building purposes of HTA. The figure is inspired by the actual structure of the Metro Map from the Russian city of Moscow and designed using ConceptDraw^®^ Pro 11 software (CS Odessa Corporation)

The “HTA Metro Map” (Figure 1 (Fig. 1)) is similar to a traffic plan map, which is basically a set of lines that cross each other, share intersection points and are concentrated in the central zone. Conceptually, these maps describe a complex network with interconnections as peculiarity. By interpreting lines as professionals working areas in the healthcare sector, the starting station is the beginning of their educational pathway and the subsequent stations are particular skills and attitudes of their profession. The multidimensional approach is graphically represented by the levels of depth of different stations, with clinical and economic aspects being within the easy reach of all professionals while others, such as organisational, ethical, legal, environmental and social aspects being much more complex to evaluate and therefore, are on the outside. The map also highlights the levels of care represented as city areas, from the central “hospital area” to the external social periphery. Additionally, the overall traffic organisation is representing the transdisciplinary coordination and exchange to assure the traffic is in time.

The structure of the HTA Metro Map is not random, it is inspired by the actual structure of the metro map from the Russian city of Moscow [11] and on the ideas of how many different kind of services have to work together to be able to serve with this kind of complex infrastructure on local, city, regional and national levels.

The concepts within the model could be identified and implemented based on the needs of users, experts and non-experts, to describe processes, needed knowledge and needed description of the area where the decision is to be done.

### At first sight: complexity

The first sight of the Metro Map, as a whole, provides the level of complexity within HTA. The complexity stems from the collaborative work of many professionals who need to communicate with each other on different topics despite having different backgrounds. Multidisciplinarity is a valuable and crucial asset within the healthcare decision making process for shared objectives like the efficient use of health technologies. Also, all professionals employed in the healthcare sector should be able to contribute without meeting hierarchically classified positions. Conceptually other professionals, even if not primarily involved in healthcare, can influence health choices indirectly. The complexity of the HTA system should be able to consider every aspect of the technology: from the easily identifiable to the more difficult ones. 

### Many lines: multidisciplinarity/transdisciplinarity

The lines represent the main actors involved in the assessment processes. The presence of 10 coloured lines, which cross each other proceeding towards the central area, creates the idea of essential professional collaboration. Each line starts far away from downtown, where most lines cross, like any professional who begins his/her educational career working on its bases. 

Often, the basic skills of a professional training are similar in the first period, but the approach to the matter may vary substantially.

The set of knowledge, skills, and attitudes builds the professional, depicted as a single line in the map; the multidisciplinary and multidimensional approach, ensured by a plurality of professionals, builds the HTA process in all the complexity of the networking interactions.

In conclusion, the figure represents just 10 main lines. We could imagine other types of transport and therefore, actors of the system that, although not always clearly identified, could play a key role in the decision-making process.

The transdisciplinary aspect is represented by the concept of traffic at the same time on all lines. Any line could function individually, but would represent a partial analysis or the whole problem, increasing the likelihood of bias in the separate use of its conclusions to make decisions. 

### Connections and crossings: interprofessional/interpersonal collaborations

Each station represents a competence or an attitude and there are single, double, triple, and quadruple connections in the map, able to connect different lines (shared skills or knowledge by different professionals). The concept behind this graphic representation is the interprofessional and interpersonal collaboration required by the HTA process. So here the different actors and groups are coming together to see whether the traffic can be synchronized again in case there has been a delay for any reason. 

In reality, the potential for sharing and cooperation between professionals and people is virtually endless.

It is important to highlight that only one or a few professionals will never be enough to consider all aspects of a healthcare technology. Only sustained cooperation between professionals and other related stakeholders including the willingness to discuss on each topic will then make shared decisions feasible, especially to ensure that the future use of any technology will be effective as the theoretical results assumed in the initial assessment of that health technology. 

### Stations: knowledge, skills, experiences and attitudes

Within the Metro Map, each line contains a number of stops that represent professional skills and knowledge. All knowledge shared by different lines must be seen as related to similar scientific/social background skills based on common or at least similar terminology and a similar kind of scientific methods and understanding. 

However, different professionals involved in HTA may sometimes lead to conflicting evaluations and outcomes, as during ethical evaluations that are often difficult to manage.

Every professional needs to be trained not only by increasing knowledge and developing specific skills and experiences (formerly competencies), but also collaborating with other professionals through their attitudes, especially in complex areas such as the ethical or social ones.

Within the lines anyone can see that different kind of specific topics, having the same name in different lines are occurring, but are handled first of all within the context of the specific profession. Also at crossing-line stations other professionals can join to support the “decision traveller” on its way to the next station and to clarify topic related issues.

### Linear lines: HTA stakeholders 

Depending on the nature of a health technology, on the decision background and on the questions which should be answered, every line can vary and can even be adapted. If urgent decisions are needed, stations could be skipped when they are not required. By this, due to different needs, the professional skills on each line can be brought in accordance to the traffic needs. E.g. if a specific implementation knowledge regarding a technology within an entire national health care system is needed, all stations should be used. Conversely, for a common transnational project some of the stations could be skipped due to the fact that there is no common level of health services provision in those nations. Thus, this would need to be added later within the national adaptation process (context tailoring). On the other side, common basic medical knowledge, compiled at the transnational level, can lead to the issue that during the national adaptation process some stations related to this knowledge can be skipped, making the process itself more efficient. Everyone should bear in mind that this is only possible if the systems are alike and thus, the national guidelines follow the internationally obtained information. Otherwise, the acceptance of this knowledge and the trust on the decision-made would be low or non-existing. 

### Circular line: patients/citizens

While linear lines are representing the different professions or disciplines and their skills required to assess and thus, make-decisions and manage patients, the circular line concept is the core of the system and is connected to most of lines on the map. Patients and citizens require information from different sources in terms of building up their discourse and being represented in the decisions made (shared and informed decisions); they need to inform and share with the community their worries, needs and outcomes of interest. This perspective shows the importance of having well-informed citizens and patients, from many sides, in order to create a fairly structured system, that is transparent and responsive to the claims of the main actors in terms of health and social system services. Patients and citizens that do not receive: on–time, sufficient, well-structured and comprehensive information around the conditions they or the systems are confronting will provide partial, biased, socially unacceptable and unethical views of the problem. Furthermore, their views will contribute to increasing the inequity of health and social provision by promoting evidences without considering the many sides and dimensions of the system and its management.

### Zones: from social, to community and hospital level of care

All lines not only connect different stakeholders and professions, but also different areas of a healthcare system or (by connecting also train stations) regions and nations. 

The zones represent the need for integration from the perspective of patient-centred systems. That means there are boundaries in terms of care, from acute to community and social care. However, these boundaries need to be replaced by an integrative and integral system that coordinates and informs people about different issues in order to provide the best solution to the individual patient. Each patient will have different itineraries including different zones. Hospital, community and social care will continue defining their services, but knowing with whom to communicate with in order to ensure continuity of care, to benefit from transdisciplinarity and to reduce inefficiencies. The concept of zones is part of the health forming process as well as the coordination and inclusion of related social and professional groups.

At the same time, other zones have the chance to use the metro lines to connect with other areas. The potential exchange of experiences and opinions as well as facts and background, can lead to effective decisions. When individual problems exceed the boundaries or limits of a single metro map the crossing of the border is necessary; e.g. big ticket technologies that require bigger populations in terms of efficiency or rare-diseases that require bigger geographical areas (referral sites) to promote a size that grants safety, effectiveness and comprehensive economy of scale.

### Elevator: different dimensions of HTA analysis (profoundness of the analysis)

According to the definition, HTA is not only a multidisciplinary but also a transdisciplinary political analysis to inform decision makers. Moreover, it requires the analysis of different dimensions that define the direct and indirect consequences of implementing, using or deleting a technology and in comparison with others. Within those dimensions, we could find, among others: health aspects such as safety and efficacy/effectiveness, economic aspects, organisational aspects, ethical issues, legal aspects, environmental and social issues. The elevator tries to represent these different levels of analysis (profoundness) from a minimum and core analysis to a tight and accurate analysis that embraces all those mentioned aspects [12]. The latter can not be easily shared within different contexts without considering the differences among them. That is the reason why, the same technology with different outcomes in mind and applied to different systems could lead to different decisions. Accordingly, someone that reads a HTA report should be aware of the level or profoundness of the analysis, in order to consider to which extent the conclusions and recommendations could be applicable in the context of interest without amendments or adaptation or even further new assessment in terms of ensuring its validity and applicability. The elevator gives the possibility of following only a base-line or of assessing more interconnections leading to inclusion of more different dimensions to the HTA analysis. The level of profoundness could also define the time required for doing this analysis, the further we go, the longer the process will be; thus the process will be tailored better to the context. This is applicable to regional analysis, in which the profoundness and the characteristics of the systems will define the evidence or evidences that could be shared among HTA doers. That is internal validity defined from the method and external validity or applicability defined from the degree the health care systems differences affect the direct or adapted adoption of the analysis.

### The visual perspective: the city centre and a view beyond the boundaries 

The Metro Map figure also tries to represent the indirect consequences that individual decisions might have on the final decision applied to a technology. Any delay in providing information around a single technology (access to technology delays) or any decision that is based on insufficient data that leads to a rapid decision, provoke a cascade of consequences. For example how the budget will be used or how new technologies knocking at the door will be considered. Any investment requires a cautious consideration as it will influence the amount of budget possibly invested elsewhere. The Metro Map is attractive from another point of view. Trains can be delayed (access to treatments or budget constrains) or the coordinator of the metro could give priority access to different lines (adaptive pathways or decisions on priorities in health). Not all health systems have the same priorities or goals. These might influence what is going to be assessed and moreover, what is going to be implemented or deleted from the systems.

### The temporal perspective: decisions and future impact

In the long term the Metro Map represents the healthcare system at a whole. To which extent are the lines renewed or meaning innovations implemented? Here the acceptance of the HTA user, the passenger of the metro, can also be taken into account and the use of the lines by the user could be consider the success of HTA analysis for decision-making. Likewise, the healthcare systems could be anchored to their own complacency around previous successful achievements without considering the requirement of continuous improvement to ensure the best possible outcomes at the time being. HTA plays a crucial role here by providing the data for possible changes and solutions. Furthermore, HTA analysis should reflect and surface the requirements and characteristics of health organisations and needs of patients, professionals and citizens; any decision will affect both short and long term conditions. 

## Conclusions

The importance of HTA for increasing efficiency in healthcare decisions is well acknowledged.

The model of a “Metro Map” provides a multidimensional and transdisciplinary conceptual approach for understanding the complexity of HTA. Actors, interactions, background and needs are visualized for easy guidance of all actors in HTA, from scientist to decision maker. 

The map is not designed to provide an accurate depiction of the complexity. In that respect, the map is an approach to visualize the HTA-complexity in a simplified model. The strength of the Metro Map is the ability to identify the relative position and expertise of different stakeholders, the connections among them and the required interactions for better and efficient decisions with the patient in the centre of the healthcare system. The map also provides a good representation of the overall system from whatever position it is viewed. Another advantage is that it can be altered and adjusted as needed. Each metro station could have a different name and could be in a different position in each line. To illustrate a more complex system, it may be possible to add more lines and stations; even other metro maps (for complex technologies, rare conditions and required higher competences). Thus, it is clear that the idea is not to provide a one-size-fits-all tool. The map is a flexible tool useful to capture the complexity of the healthcare system which at the same time is adaptable for local, national or international purposes. This will assist different stakeholders to build network capacity, pool existing resources, and to develop a more holistic vision that, hopefully, will result in a sustainable (efficient) and collaborative decision making process.

## Notes

### Author contributions

All authors contributed equally to this work. 

### Competing interests

The authors declare that they have no competing interests.
